# Influences of Plant Species, Season and Location on Leaf Endophytic Bacterial Communities of Non-Cultivated Plants

**DOI:** 10.1371/journal.pone.0150895

**Published:** 2016-03-14

**Authors:** Tao Ding, Ulrich Melcher

**Affiliations:** Department of Biochemistry and Molecular Biology, Oklahoma State University, Stillwater, Oklahoma, United States of America; Free University of Bozen/Bolzano, ITALY

## Abstract

Bacteria are known to be associated endophytically with plants. Research on endophytic bacteria has identified their importance in food safety, agricultural production and phytoremediation. However, the diversity of endophytic bacterial communities and the forces that shape their compositions in non-cultivated plants are largely uncharacterized. In this study, we explored the diversity, community structure, and dynamics of endophytic bacteria in different plant species in the Tallgrass Prairie Preserve of northern Oklahoma, USA. High throughput sequencing of amplified segments of bacterial rDNA from 81 samples collected at four sampling times from five plant species at four locations identified 335 distinct OTUs at 97% sequence similarity, representing 16 phyla. *Proteobacteria* was the dominant phylum in the communities, followed by the phyla *Bacteriodetes* and *Actinobacteria*. Bacteria from four classes of *Proteobacteria* were detected with *Alphaproteobacteria* as the dominant class. Analysis of molecular variance revealed that host plant species and collecting date had significant influences on the compositions of the leaf endophytic bacterial communities. The proportion of *Alphaproteobacteria* was much higher in the communities from *Asclepias viridis* than from other plant species and differed from month to month. The most dominant bacterial groups identified in LDA Effect Size analysis showed host-specific patterns, indicating mutual selection between host plants and endophytic bacteria and that leaf endophytic bacterial compositions were dynamic, varying with the host plant’s growing season in three distinct patterns. In summary, next generation sequencing has revealed variations in the taxonomic compositions of leaf endophytic bacterial communities dependent primarily on the nature of the plant host species.

## Introduction

Endophytic bacteria are harbored inside healthy plant tissues but do not lead to pathogenic reactions [1], and play important roles in phytoremediation[2–5], biological control against insects or pathogenic microorganisms [6, 7], and plant growth promotion [8–10]. Endophytic bacteria may also be pathogenic to other plants, animals, especially cattle, and human beings [11–14].

Endophytic bacteria can be divided into two types according to where they are harbored on the host plants: root endophytic bacteria, which are a subset of rhizosphere bacteria, and leaf endophytic bacteria, which are a subset of phyllosphere bacteria. Traditional microbiological approaches have been widely applied in endophytic bacterial research with a special emphasis on the roots of cultivated plants including sugarcane [15, 16], ginseng [17, 18] and potato [19] to explore plant growth-promoting bacteria [20] because of their large contribution to plant nutrient intake as well as to the high diversity of soil bacteria. The development of next generation sequencing (NGS) has revolutionized microbial community research: the Human Microbiome Project (HMP) [21, 22] utilized NGS techniques to characterize comprehensively the human microbiome and to study its role in human health and disease and the Earth Microbiome Project (EMP) attempted to characterize global microbial diversity [23]. Similarly, high throughput sequencing techniques have greatly promoted research on endophytic bacterial communities: root endophytic bacterial microbiota have been profiled in both model plants and cultivated crops using NGS techniques.

In contrast to research on root endophytic bacteria, leaf endophytic bacteria have been less well studied. Leaf endophytic bacteria are a subset of phyllosphere bacteria, along with bacteria living on the surface of the leaves, termed “epiphytic bacteria” [24, 25]. Leaf endophytic bacteria live inside the leaves and may maintain an endophytic symbiotic relation with the host plants. The distinction suggests that leaf endophytic bacteria are more involved in plant biology than leaf epiphytic bacteria and thus their compositions and distributions are more likely to be determined by the nature of the host plants. Actually the endophytic colonization by epiphytic bacteria indicates that these microbes have passed the firewall against the host plant’s innate immunity, which can terminate the microbial growth by detecting the microbe-associated molecular patterns (MAMPs) [26].

Yang et al. in 2001 first used a culture-independent methodology to reveal that the complexities of phyllosphere bacterial communities are far beyond previous estimation [27]. Following that report, a few studies characterized the phyllosphere bacterial communities in model [28] and cultivated plants [29–31]. Being the major part of the plant surface, the leaf surfaces harbored a great diversity of phyllosphere microorganisms [25, 28]. Knief et al. found that the host plant species and locations are important determinants of the Methylobacterium community compostion [32]. Closely associated with phyllosphere microbiota, leaf endophytic bacterial communities also have a great diversity. By studying model plants, Bodenhausen et al. found that leaf and root endophytic bacterial communities have similar diversity, richness and eveness, but their compostions are significantly different [28]. In contrast to our current knowledge about phyllosphere bacterial communities, information on the diversity of leaf endophytic bacteria is scarce, especially on non-cultivated plants.

Previously, by extensive rising of the leaf surface to remove epiphytic microbiota, we applied Terminal Restriction Fragment Length Polymorphism (T-RFLP) to study leaf endophytic bacterial communities of non-cultivated plants of the Tallgrass Prairie Preserve (TPP) of Osage Co., Oklahoma [33]. The T-RFLP study suggested a three-factor model for description of variation in leaf endophytic bacterial communities with host plant species, collection dates and sampling sites all having significant impacts. We also identified some dominant T-RFs, representing specific bacterial groups. This provided us a quick and efficient method to examine the variation of leaf endophytic bacterial communities.

In the present study, we applied high-throughput tagged pyrosequencing [34] of the 16S ribosomal RNA genes of the leaf endophytic bacterial communities from non-cultivated plants of five different species, to profile the compositions of leaf endophytic bacterial communities, to evaluate the community diversity and to determine the factors that significantly shape the communities. Specifically, we sought to define the dominant taxa at different levels in the leaf endophytic bacterial community, to compare the bacterial communities hosted in plants of different species or collected at different times to find the key bacterial taxa which characterize the differences, and thus validate the model we proposed in the T-RFLP study regarding the factors determining bacterial community structure and diversity in such communities.

## Methods and Materials

### Plant sampling & DNA extraction

Leaf samples from plants of five species, *Ambrosia psilostachaya* DC., *Asclepias viridis* Walt., *Panicum virgatum* L., *Sorghastrum nutans* (L.) Nash and *Ruellia humilis* Nutt., in the TPP were collected at four different sites in each month from May to August, 2010 as described previously [35]. Permission to collect samples in the TPP, Osage Co. OK, owned by The Nature Conservancy, was granted by Dr. Robert G. Hamilton, Director. The five species were chosen on the basis of their wide distribution among study sites in the TPP and their abundance at these sites at all of the sampling times. In each of the four months, one sample of each species was collected at each of the four locations resulting in a total of 81 collected samples.

To eliminate a contribution of epiphytic bacteria to the analysis of leaf endophytic bacteria, the surfaces of plant samples were washed first with tap water and then with 75% ethanol, and finally rinsed with running distilled water. The efficacy of this treatment was validated by plating the rinse water on nutrient agar. All leaves of each plant were ground in liquid nitrogen and 0.1 g aliquots were used for total DNA (including plant and bacterial DNA) extraction as described previously [35].

### PCR amplification and pyrosequencing

For DNA from each plant sample, PCR was conducted using a pair of primers that incorporated a unique barcode [36] so that the sequences of the PCR amplicons could be assigned to the correct samples based on the barcodes. To avoid PCR amplification of plant plastid DNA, which is highly similar to bacterial 16S rDNA, a pair of primers, 799F and 1492R, which do not amplify plastid rDNA [37], was used to amplify the bacterial and plant mitochondrial rDNA. The resulting amplicon fragments covered several variable regions of 16S rDNA including V5 to V8 and part of V4 [38]. The forward primers (5’- CGTATCGCCTCCCTCGCGCCATCAGX8CAAACMGGATTAGATACCCKG-3’) contained the 454 sequencing primer A, the barcode [36] represented by X8, a 2-base linker sequence CA, and the primer 799F. The reverse primer (5’- CTATGCGCCTTGCCAGCCCGCTCAGTCGGCTACCTTGTTACGACTT-3’) contained the 454 sequencing primer B, a 2-base linker sequence TC, and the primer 1492R. PCR products were separated by electrophoresis in 2% agarose to remove amplicons of plant mitochondrial DNA. Following PCR and electrophoresis, bacterial 16S amplicons (approximately 700 bp) were recovered from the gels to exclude the plant mitochondrial 16S amplicons (approximately 1kb) from downstream analysis. The bacterial amplicon DNA was purified from the gel using Qiaquick Gel Purification Kit (Qiagen, Hilden, Germany). An equimolar mix of purified amplicon libraries from all plant samples was made and prepared for pyrosequencing by emulsion bead PCR using the recommended Lib-A kit and protocol (454 Life Sciences, Branford CT). Sequencing using the Genome Sequencer Junior Titanium Series platform (Roche, Indianapolis IN) was conducted at the Oklahoma State University Array and Bioinformatics Core Facility. Sequencing data and plant metadata have been deposited in the Sequence Read Archive of the National Center for Biotechnology Information in Project PRJNA201047.

### Sequence analysis

The raw sequencing data were generated as sff files from the Roche 454 Junior sequencer and were analyzed using the 16S rRNA gene sequence curation pipeline implemented in mothur software [39]. Briefly, raw sequences, fasta and quality files were extracted from sff files, and any sequence that had at least one mismatch to the barcode or two mismatches to the primer sequence were removed. Any sequences that contained eight nucleotide or longer homopolymers or an ambiguous base call were also culled. Trimmed sequencing reads were de-noised using the PyroNoise algorithm [40] and then aligned against a customized SILVA database reference alignment [41], followed by the detection and removal of chimaeric sequences using a de novo Uchime algorithm [42]. The sequences were then clustered using the furthest neighbor clustering algorithm to build operational taxonomic units [OTUs] at a cutoff of 97% sequence similarity, and then classified using the naive Bayesian Classifier which had been trained against a customized RDP training set [43], so that only the bacterial-origin sequences remained. Finally we obtained a table reporting the counts of observation times of an OTU in each sample. OTUs that had a total count less than 10 were removed and all samples were sub-sampled to 200 reads for downstream analysis. Alpha diversity indices were calculated as Shannon Index, implemented in mothur. Statistical analysis was performed using Tukey’s HSD test in the R program. LEFSe analysis was performed based on an algorithm proposed by Segata N. et al [44] implemented in mothur.

## Results

### Sequencing summary and composition of endophytic bacterial communities

**Fig 1:**
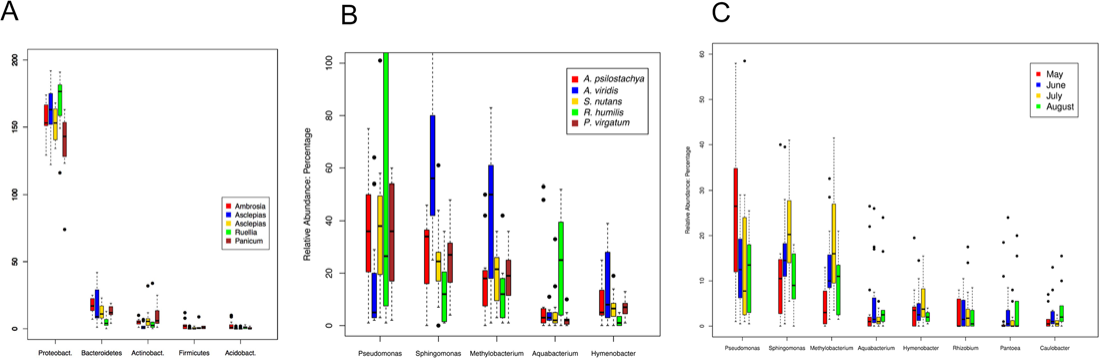
Variation of relative abundances of dominant bacterial groups with the host plant species and sampling months. Relative abundances of the dominant phyla in the leaf endophytic bacterial communities from different host plants (A); Relative abundances of the dominant bacterial genera across different host plant species (B) and across different sampling months (C).

We obtained 64,952 raw sequence reads from high throughput 454 sequencing. Using the 16S rRNA gene sequence curation pipeline resulted in an OTU table reporting 335 OTUs from 55 plant samples representing five plant species. The phylum *Proteobacteria* dominated (85.42%) the leaf endophytic bacterial communities on the samples collected from the 5 species of plants (Fig 1A), followed by *Bacteroidetes* (7.21%) and *Actinobacteria* (3.21%), and then by *Firmicutes* (0.63%) and *Acidobacteria* (0.59%).

At the class level, three classes from *Proteobacteria* (*Alpha-proteobacteria* (44.68%), *Gamma-proteobacteria* (28.88%) and *Beta-proteobacteria* (10.87%)) were in dominant positions. In addition, the class of *Sphingobacteria* (5.68%) from the phylum *Bacteriodetes* and the class *Actinobacteria* (3.21%) of the phylum *Actinobacteria* were also abundant.

At the genus-level, the genera of *Pseudomonas* (19.42%), *Sphingomonas* (18.79%) and *Methylobacterium* (12.83%) were the most dominant. In addition, *Aquabacterium* (4.58%), *Hymenobacter* (4.34%) and *Rhizobium* (3.24%), *Pantoea* (2.86%) and *Caulobacter* (2.27%) were also abundant genera with relative abundances greater than 2%.

### Dominant microbial groups in endophytic bacterial communities

The species of host plants has been shown to be a major factor in determining the endophytic bacterial communities [35]. The sequencing results also revealed that the dominant phylotypes varied in the endophytic bacterial communities among plants of different species (Fig 1B). Although the genus *Pseudomonas* was the most abundant genus across all microbial communities (Fig 1A), its relative abundance in *A*. *viridis* is significantly lower than in other plant species. In contrast, the two other dominant phylotypes, *Sphingomonas* and *Methylobacterium*, were significantly more abundant in *A*. *viridis* than in other plant species. Similarly the phylotype *Aquabacterium* was significantly more abundant in *R*. *humilis* than in other plant species. These discoveries indicate that the relative abundances of specific endophytic bacteria are associated with the type of the host plant that harbors the microbial community.

The dominant genera also showed a dynamic pattern, varying across the whole plant-growing season (Fig 1C). The most dominant genus, *Pseudomonas*, became less abundant from May to July, followed by a come-back increase in August. In contrast, the other two dominant genera *Sphingomonas* and *Methylobacterium*, turned more abundant from May to July, followed by a drop in relative abundance in August. These dynamic variations suggested unique and characteristic growing patterns of endophytic bacteria, patterns which are sensitive to environmental changes associated with seasonal variables.

### Diversity of the endophytic bacterial communities

We also calculated the alpha diversity of each endophytic bacterial community. Alpha diversity is the within sample taxonomic diversity, which contains the information of both the richness of a sample and the evenness of the organisms’ abundance distribution [45]. Here the alpha diversity was quantified by the Shannon index [46].

**Fig 2:**
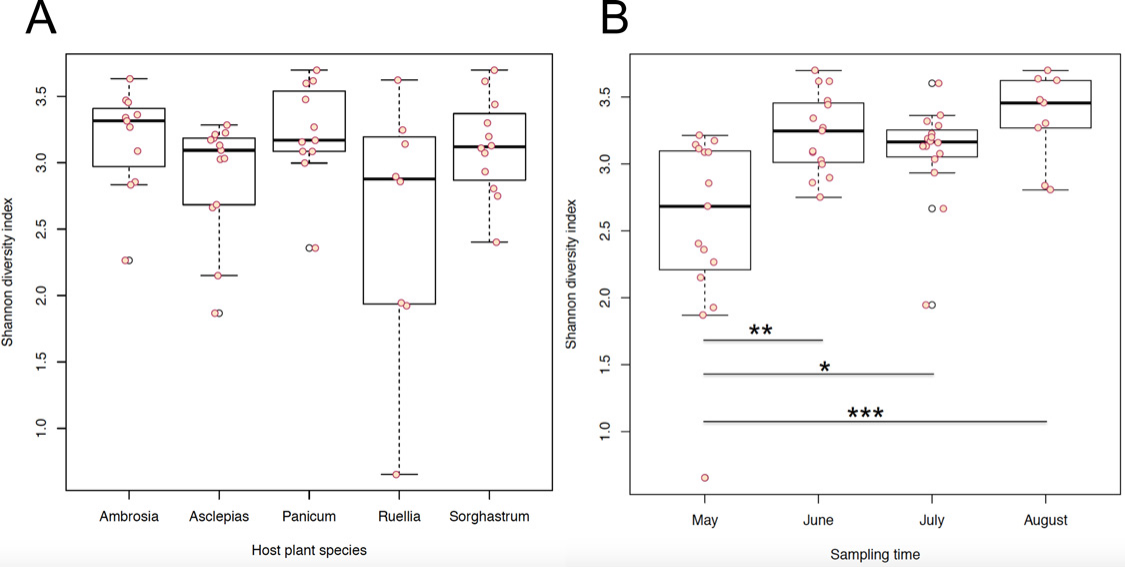
Alpha diversities of each endophytic bacterial community grouped by host plant species (A) and by sampling time (B).

Fig 2A shows the alpha diversity of each endophytic bacterial community grouped by host plant species. Although the indices showed some difference across multiple host species, no significant difference was observed. However, grouping these diversity indices according to time points (Fig 2B), revealed that the diversity of bacterial communities varied from one month to another: the diversity index in May was significantly lower than in June (p <0.01), July (p<0.05) and August (p<0.001). This indicated that the endophytic bacterial communities become more diverse as the host plants emerged into an intense growing season.

Analysis of molecular variance (AMOVA) [47] was also performed to test the significance differences of the diversity between different groups. AMOVA has been widely used in population genetics to test if the genetic diversity within each population is significantly different from that of the pooled population. We tested three categories of factors: the host plant species, collection time points and locations. The results indicated that, despite the finding of no significant differences of diversity among different collection locations, significant differences of diversity were observed from endophytic microbial samples collected from host plants of different species, or from different time points (months) (p-value < 0.001). This result is consistent with the previous T-RFLP study, again showing that the host plant species and sampling time points are two factors that have significant impacts on the distribution of endophytic bacteria. The AMOVA analysis on collection time points (Table 1) shows that diversity of the communities collected in May was significantly different from June (p-value < 0.05), July (p-value < 0.001) and August (p-value < 0.05), while analysis on host plant species (Table 2) shows the diversity of bacterial communities from host plants of different species are distinct from each other.

**Table 1:** AMOVA analysis of differences in diversity of endophytic bacteria in different. sampling months.

*p*-value	May	June	July
June	0.024		
July	< 0.001	0.068	
August	0.016	0.373	0.02

Comparing all four months: *p* = 0.001

**Table 2:** AMOVA analysis of differences in diversity of endophytic bacteria in different host plant species.

*p*-value	*A*. *psilostachya*	*A*. *viridis*	*P*. *virgatum*	*S*. *nutans*
*A*. *viridis*	< 0.001			
*P*. *virgatum*	0.016	< 0.001		
*S*. *nutans*	0.106	< 0.001	0.962	
*R*. *humilis*	0.019	< 0.001	< 0.001	< 0.001

Comparing all five species: *p* < 0.001.

### Defining the core of the leaf endophytic bacterial community

Among the 355 defined OTUs across all the samples, a core leaf endophytic microbiome was observed. Although there are no OTUs that exist in all 55 samples after the subsampling and data filtering, our data reveals that 4 OTUs commonly exist in at least 33 samples (60% of all samples) with a minimum relative abundance of 1%: OTU 2476 and OTU 1327, both from the genus *Sphingomonas*, and OTU 2857 and OTU 3184, both from the genus *Methylobacterium*. It is worth noting that OTU 2476 is a highly abundant core microbe in the leaf endophytic communities: its relative abundance is greater than 2% in at least 36 samples (65% of the total) and greater than 5% in at least 27 samples (50% of the total).

Core leaf endophytic microbiome for each species of host plants were also explored. In *A*. *psilostachaya*, a core of 10 OTUs (with minimum relative abundance 1%) exists in at least 50% of all samples, and OTU 980, from the genus *Pseudomonas*, is the most abundant core microbe (relative abundance greater than 7% in all samples). Similarly, in *P*. *virgatum* and *S*. *nutans*, a core of 9 and 13 OTUs were found, and OTU 980 was the most abundant core microbe. In *A*. *viridis*, a different core of 11 OTUs were found in at least 50% of all samples, and the most abundant core microbes were OTU 1327 and OTU 2857 (relative abundance greater than 7% in all samples). In *R*. *humilis*, a core of 8 OTUs were found, and the most abundant core microbes were OTU 2245, from the genus *Pseudomonas*, and OTU 2528, from the genus *Aquabacterium*.

We also analyzed the core leaf endophytic microbiome for each time point when we collected the samples. In May, at the level of minimum relative abundance 1%, only OTU 2245 existed in at least 50% of all samples, and this OTU had a minimum relative abundance of 7%. In June, a core of 8 OTUs was found with minimum relative abundance 1% in at least 50% of all samples, and the most abundant core microbes were OTU 980, and OTU 2476. In July, a core of 9 OTUs were found, and the most abundant core microbes are OTU 1327 and OTU 2476. In August, a core of 10 OTUs were found and the most abundant core microbe is OTU 980.

### Discovery of microbial groups that characterize the differences among varied host plants and multiple time points

Analysis of endophytic bacterial communities using T-RFLP revealed some important T-RFs, which represent specific bacterial groups, and found that host plant species and sampling times are significant factors determining the compositions of endophytic bacterial communities. The high throughput sequencing data enables discovery of biomarker microbes that characterize the differences among host plants of different species, or across multiple time points. Here we employed LDA Effect Size (LEFSe) [44] to identify biomarker microbes that characterize the differences between two or more host plant species or collecting time points. In general, LEFSe first identifies statistically different features among biological classes using the Kruskal-Wallis (KW) sum-rank and Wilcoxon rank-sum tests, and then uses Linear Discriminant Analysis (LDA) to reduce the dimension of the detected features.

**Fig 3:**
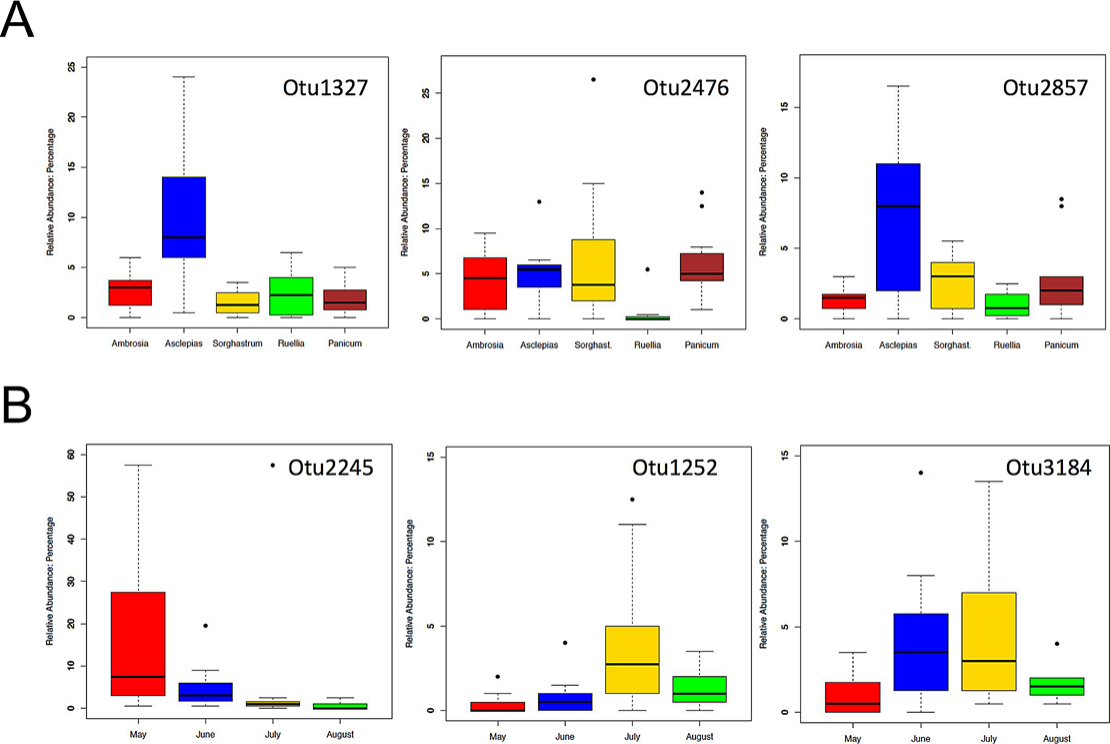
Relative abundances of the feature OTUs selected by LEfSe analysis across different host species (A) and across different sampling months (B).

Table 3 shows the result of the LEfSe analysis, discovering biomarker microbes based on the differences between endophytic bacterial communities between two or more host plant species. Five OTUs were significantly different in at least one host plant species relative to others (p<0.05). The relative abundances of OTU 1327, from the genus *Sphingomonas*, and OTU 2857, from the genus *Methylobacterium* in *A*. *viridis* (6.56% and 4.69% respectively) (Fig 3A) were significantly higher than those in other host plant species, while OTU 2476 was found by LEFSe to be significantly higher in *S*. *nutans* (4.50%) than in other species. The discoveries of these OTUs as biomarkers, indicates that the differences among the endophytic bacterial communities from diverse host plant species are not only measured by diversity index or dominant microbes, which are generally categorized as characteristics of the whole community, but also reflected in the existence and/or relative abundance of single microbe or microbial groups.

**Table 3:** LEfSe analysis identification of biomarker microbes for endophytic bacterial communities for different host plant species.

OTU	Type	LDA	p-Val	Phylum	Genus	*Ambrosia*	*Asclepias*	*Panicum*	*Ruellia*	*Sorghastrum*
Otu1327	*Asclepias*	4.701	0.0002	*Proteobacteria*	*Sphingomonas*	18.2	65.6	12.4	16.2	10.3
Otu2476	*Sorghastrum*	4.568	0.0106	*Proteobacteria*	*Sphingomonas*	27.9	31.5	42.1	5	45
Otu2857	*Asclepias*	4.592	0.0255	*Proteobacteria*	*Methylobacterium*	9.4	46.9	19.1	6.7	16.7
Otu1215	*Sorghastrum*	4.341	0.0376	*Bacteriodetes*	*Hymenobacter*	2.1	7.9	6.7	1.2	7.5
Otu2934	*Asclepias*	4.395	0.0483	*Bacteriodetes*	*Hymenobacter*	16.7	28.5	8.5	2.9	12.2
Otu1088	*Ambrosia*	4.359	0.0668	*Proteobacteria*	*Pseudomonas*	21.5	2.1	7.6	2.5	6.7
Otu1252	*Asclepias*	4.358	0.0785	*Proteobacteria*	*Methylobacterium*	3	26.4	6.1	5.4	9.2
Otu514	*Asclepias*	4.368	0.0994	*Proteobacteria*	*Sphingomonas*	10.9	20.3	3.9	3.3	2.2
Otu2528	*Ruellia*	4.745	0.3059	*Proteobacteria*	*Aquabacterium*	37.3	11.3	7.9	77.5	18.6
Otu2245	*Ruellia*	5.139	0.3455	*Proteobacteria*	*Pseudomonas*	40.9	18.2	24.8	192.9	35.6
Otu3184	*Asclepias*	4.331	0.4293	*Proteobacteria*	*Methylobacterium*	30.6	37.2	13.6	14.6	9.2

Table 4 shows the result of LEfSe analysis of the biomarker microbes for endophytic bacterial communities between two or more sampling sampling months. Five OTUs were significantly different in at least one month from others (p<0.05). One of the most abundant OTUs, OTU 2245, from the genus *Pseudomonas*, was significantly more abundant in May than in other months: its relative abundance in May (12.8%) was even more than the sum of its relative abundances in the other three months (Fig 3B). Two other OTUs, OTU 1252 and OTU 3184, both from the genus *Methylobacterium*, are, respectively, more abundant in July and June. The varied abundance of these biomarker OTUs in different months shows that microbes can have their own variation independent of the whole community, and that the dynamics of single microbial groups, as part of the dynamics of the whole bacterial community, are associated with the dynamics of the host plants.

**Table 4:** LEfSe analysis identification of biomarker microbes for endophytic bacterial communities for different months of sample collection.

OTU	Type	LDA	pValue	Phylum	Genus	May	June	July	August
Otu2245	May	5.042	0	*Proteobacteria*	*Pseudomonas*	12.8	3.24	3.02	0.44
Otu1252	July	4.302	0.0004	*Proteobacteria*	*Methylobacterium*	0.27	0.49	2.54	0.85
Otu3184	June	4.38	0.0027	*Proteobacteria*	*Methylobacterium*	0.64	3.18	3.27	1.11
Otu2476	July	4.573	0.0038	*Proteobacteria*	*Sphingomonas*	1.2	3.04	5.6	2.52
Otu1088	August	4.203	0.0083	*Proteobacteria*	*Pseudomonas*	0.11	0.62	0.96	2.04
Otu1333	August	4.24	0.0505	*Proteobacteria*	*Caulobacter*	1.02	1.62	0.42	2.52
Otu2857	July	4.346	0.0661	*Proteobacteria*	*Methylobacterium*	1.07	1.98	3.6	1.59
Otu1709	July	3.94	0.0736	*Proteobacteria*	Unclassified	0.22	0.56	0.83	0.41
Otu1327	July	4.345	0.085	*Proteobacteria*	*Sphingomonas*	2.22	2.11	4.19	1.37
Otu2528	August	4.408	0.1715	*Proteobacteria*	*Aquabacterium*	2.78	3.73	0.96	3.96
Otu2934	July	4.12	0.188	*Bacteroidetes*	*Hymenobacter*	1.6	1.4	1.96	0.59
Otu1724	August	4.087	0.3628	*Proteobacteria*	Unclassified	0.78	1.24	1.56	1.85
Otu514	June	4.078	0.3672	*Proteobacteria*	*Sphingomonas*	0.76	1.64	0.58	0.3
Otu1863	June	3.894	0.5506	*Actinobacteria*	*Curtobacterium*	0.24	0.98	0.92	0.56
Otu1215	July	3.873	0.7169	*Bacteroidetes*	*Hymenobacter*	0.49	0.33	0.81	0.52
Otu2077	July	3.862	0.7716	*Proteobacteria*	*Sphingomonas*	0.58	0.82	0.94	0.44
Otu980	May	4.275	0.8057	*Proteobacteria*	*Pseudomonas*	5.82	4.91	5.62	5.78
Otu2573	July	4.036	0.8927	*Proteobacteria*	*Rhizobium*	1.58	1.96	2.19	1.26
Otu269	July	3.767	0.9565	*Proteobacteria*	*Sphingomonas*	0.38	0.38	0.42	0.41

## Discussion

The present study is the first to apply next generation sequencing on the leaf endophytic bacterial communities harbored on multiple species of non-cultivated plants. After denoising sequencing errors and chimeras, we obtained 335 97% sequence similarity OTUs from the sequencing of a 700bp fragment of 16S ribosomal DNA gene, representing 16 bacterial phyla, 31 classes, 50 orders, 110 families and 221 genera. Plant leaves cover a significant proportion of the land area of the earth, and thus also provide habitats for colonization of a high diversity of microorganisms. This study provided a survey of the distribution of leaf endophytic bacteria harbored in non-cultivated plants, making an important contribution to the bacterial diversity research of the ecosphere.

Davide Burgarelli et al. studied Arabidopsis root-inhabiting bacterial microbiota [26] and found it is dominated by three phyla, *Proteobacteria*, *Bacteriodetes* and *Actinobacteria*. Later, Bodenhausen et al. compared bacterial communities that are associated with leaves and roots of *Arabidopsis thaliana* [28], and found that the same three phyla noted above are the most abundant phyla in both types of bacterial communities. In the present study, we found similar results in the five non-model plant species studied, consistent with these three phyla being the most dominant in endophytic bacterial communities from both roots and leaves across multiple species of host plants.

Profiling the microbial communities based on the sequencing results allowed us to calculate the alpha diversity of each community. Bodenhausen et al. compared the diversity of root and leaf associated microbiota on *Arabidopsis thaliana* model plants [28] and found that although their compositions are completely different, they have similar richness, diversity and evenness. Hunter et al. studied phyllosphere bacterial communities collected from 26 Lettuce accessions and calculated the alpha diversities as Shannon indices [29], and found that the diversity of phyllosphere microbiota was significantly varied among different accessions and concluded that host plant leaf properties determine phyllosphere bacterial diversity. In the present study, we compared the diversity of endophytic bacterial communities collected from different host plants and from different time points. Although we did not observe significant differences in alpha diversity [calculated as Inverse Simpson index) from the five plant species tested, we observed a significant difference of diversity between communities that were collected from different months: the diversity of the community collected in May was significantly lower than those collected in later months (June, July and August]. The core microbiome analysis also suggested that the May microbiome was dominated solely by OTU 2245 and no multi-microbe core microbiome existed. This discovery, together with the determining effects of leaf property proposed by Hunter et al. [29], suggested that the endophytic bacterial diversity is associated with host plant properties. After entering the flowering stage, the metabolic level of the host plants reached maximum. The sequencing data suggest that the prosperity of the host plants leads to a high diversity of associated microbial communities,

The core microbiome analysis shows that no multi-microbe core can be found in endophytic communities across different plant species and locations. This result indicates that the variations in endophytic communities are largely due to the features of host plant or sampling locations. However, microbes from the genera *Sphingomonas* and *Methylobacterium* are found to be abundant core microbes existing in most samples. Interactions between these common endophytes and their host plants should be further explored. The core microbiome for each plant species suggests that the interactions between endophytes and host plants in *A*. *psilostachya*, *P*. *virgatum* and *S*. *nutans* probably share similar features since *Pseudomonas* OTU 980 is the most abundant core microbe in all of these three species.

Here, we also performed AMOVA analysis to evaluate the differences of beta diversity of microbial communities collected from different host plant species or from varied time points. Although no significant differences were observed for communities collected from varied locations, both host plant species and collection time points have significant impacts on the beta diversities of the endophytic bacterial communities (Tables 1 and 2). This result is consistent with the conclusion we made previously with T-RFLP studies [35] that the endophytic bacterial distributions are significantly determined by the factors of host plant species and collection time points. Through AMOVA, the beta diversity of the bacterial communities collected in May was significantly smaller than those collected in other months; this discovery, together with what we found in the alpha diversity study, indicated that endophytic bacteria are affected by the temporal factors and host plant blooming phenology.

Important in comparative studies of microbial communities is what bacterial groups or taxa are responsible for differences between communities, or are the key features of a specific type of microbial communities. In this study, we employed the LEfSe algorithm and identified specific OTUs that characterized the differences of endophytic bacterial communities collected from varied host plants (Fig 3A) or collected in different months (Fig 3B).

In summary, the present study provided a comprehensive survey of leaf endophytic bacterial communities that inhabited non-cultivated plants. Host plants of diverse species with multiple collection time points and locations were included, which allowed us to compare the endophytic bacterial communities collected from different environment. Our research found that leaf surfaces provided habitats for a large diversity of endophytic bacteria, and their distribution was shaped by the host plant species and dates of collection. The communities from different host plant species or varied time differed in multiple aspects including compositions, alpha and beta diversity, dominant and characteristic bacterial groups.
